# Notes

**Published:** 1899-04

**Authors:** 


### NOTES.

Tennessee has been considering a more thorough veterinary sanitary service. Nashville will have a union abattoir, and the tag-system of inspection will be adopted.

Dr. D. C. Bennett, a graduate of Toronto, was among those engaged by our government as an inspector of the Government's livestock in our recent conflict with Spain. He was assigned to duty in Porto Rico, and charged with the care of horses and mules. He says "there is not a disease to which horseflesh is heir that is not seen here in all its various forms, and there are many that are not seen in the States at all. Mules, apparently, are much better adapted to this climate than horses, and the loss of mules is slight as compared with the loss of horses. Horses of the coarser breeds are of little value here, and the Clyde especially is almost worthless. I firmly believe that if this stock was kept here for one year there would not be a horse remaining that had a drop of Clydesdale blood in his veins. The native horse is very small, the heaviest not weighing more than eight or nine hundred pounds, but the greater majority weigh about six hundred. However, small as they are, their power of endurance is really wonderful. They are seldom shod, and almost as seldom fed grain, yet almost any one of them will carry a man over two mountains much easier and farther in a day than one of our best American horses. All heavy hauling is done with oxen, and in cattle-raising these people are strictly up-to-date. The manner in which the oxen are worked, however, is cruel in the extreme. The yokes are bound to their horns with raw-hides and they are hitched to great heavy two-wheeled carts which, aside from pulling, they are required to balance. Instead of using a whip or goad, they use a stick with a sharp piece of steel in the end, prodding them unmercifully. General Henry issued an order against this, and even furnished the natives with American yokes, but it seems they refuse to use them."

### Eleven Dairy Rules.

1. Keep the cows clean and wash the udders before milking.
2. Keep the barn clean, with walls and ceilings whitewashed; have it well lighted, ventilated, and free from dust at milking time.
3. Always make a clean toilet before commencing to milk.
4. Keep utensils clean and bright.
5. Remove the milk from the stable as soon as drawn, and strain and cool at once.
6. Never expose milk to bad odors.
7. Do not mix fresh warm milk with that which has been cooled.
8. Give the cows only good wholesome food and pure water.
9. Never add anything to milk to prevent its souring. Cleanliness and cold are the only preservatives needed.
10. Milk regularly, quickly, quietly, and thoroughly.
11. Always treat the cows kindly and never excite them by loud talking, hard driving, and abuse of any kind.

The United States Department of Agriculture has recently issued a bulletin having for its title *Thirty Poisonous Plants of the United States*. It represents the results of three years' investigation by the botanical experts of the department, whose researches covered a period of several centuries. This is not a large list, and most of the plants named therein and their dangerous qualities have been known for years. To Dr. Stalker, of the Iowa Agricultural College, Ames, Iowa, belongs the honor of discovering the poisonous nature of one of the thirty plants described in this bulletin, concerning which plant we quote the following: "The poisonous constituent is unknown, but it resides in both the leaves and the seed. Horses and sometimes cattle are killed by eating grass and meadow hay mixed with the plant; they are not poisoned so often by eating the plant in the field. Public attention was first called to the poisonous nature of the rattle-box by Dr. Stalker, of Iowa, who, in

1884, while investigating the cause of 'bottom-disease,' then prevalent among horses in Iowa, was led to believe that it was mostly if not altogether attributable to this plant. Experiments were made which proved the supposition to be correct."—*Ames Intelligencer.*

### The Best Hen.

The hen of medium size for the breed is usually the best layer. She is of active disposition, with healthy, red color in comb and wattles, and has a good appetite. By watchfulness you can select such hens, and if you will keep only such as breeders you can work your flock up to very great value. Vigor and egg-production are the "points" any farmer not up to all the requirements of the fancier can always appreciate, and these he can have by judiciously developing a rule of selection among the hens, and never keeping any cock that is not purely bred and of the breed that has first been employed in the improvement of the flock, and of individual merit. It is important, too, to know that he comes of a family noted for layers.

Hogs breed so rapidly that there is no excuse for any farmer to keep those which are of mongrel breeds. The poorest farmer can at least afford to own a thoroughbred pig and breed all his sows to it.

"A Horse-doctor." We do not know whether the medical director at Chickamaugua is justly criticised or not for his administration of that great camp, but it is worth while to say that those officers who think they are casting opprobrium upon Dr. Huidekoper by calling him a "horse-doctor," if they are not actuated by malice, display an ignorance hardly pardonable in a military man.

That he is a "horse-doctor" is precisely the thing that has given Dr. Huidekoper his high professional distinction—that is, that he was practically the first man in America to bring the authority of an educated physician of influential position to the study and practice of veterinary medicine, with the well-defined purpose of elevating it above the empiricism of the old-fashioned horse-doctor to a recognized branch of medical science.

The importance of this service has been recognized by all well-informed persons. Dr. Huidekoper graduated in medicine at the University of Pennsylvania and continued his studies abroad. He was interested in horsemanship and in military affairs, and the neglect of veterinary medicine in this country was practically impressed upon him, as it had been upon other military men and upon some wealthy owners of horses in the neighborhood of Philadelphia. With their encouragement Dr. Huidekoper went abroad and spent some years in the best veterinary schools on the Continent, taking his diploma from that at Alfort, and on his return became the head of the department of veterinary medicine established at the University, the first in America organized on a broadly scientific basis.

The influence of this work has extended not only to the care of horses, but to the treatment of the diseases of cattle and of other domestic animals and to many important branches of public sanitation and hygiene. Dr. Huidekoper was also prominent in the medical service of our National Guard up to the time of his removal to New York, where it is probable that his veterinary practice has absorbed most of his time. But the reason of his great prominence as a veterinarian is that he had developed this specialty upon the foundation of a broad medical education, so that the title of "horse-doctor" is to him a distinction and not a reproach.

Among all the civilians appointed to advanced rank in the volunteer service there was none whose experience and reputation gave better assurance of fitness for military responsibility. Whether any civilian should have been put in charge of so great a camp as that at Chickamaugua may be questionable, and whether Dr. Huidekoper has performed his duties well or ill we do not pretend to know. But his services to an important and previously neglected department of medical science have been of the highest public importance, and those to whom a reference to such services appears derogatory display an ignorance that casts doubt upon the value of their opinions.—Editorial *Philadelphia Times*, September 22, 1898.

Dr. W. W. Martin, of Philadelphia, will shortly enter the army veterinary service, and will add strength and value to our work in this direction.

The printing, postage, and clerical expenses of the Army Legislative Committee reached about two hundred and twenty-five dollars.

Spain had in Cuba one veterinary major, eleven veterinary captains, and sixty-four veterinary lieutenants. Spain purchased many horses and mules in the United States for the cavalry service in Cuba.

